# Supplementary material for: Pt size-dependent reverse oxygen spillover on Sn-doped Pt/TiO2 for CO oxidation
Source: Nat Commun. 2026 Mar 3;17:3380. doi: 10.1038/s41467-026-69327-x (PMC13066629; doi:10.1038/s41467-026-69327-x)
Supplement: Supplementary file 1 — Supplementary Information [file 41467_2026_69327_MOESM1_ESM.pdf]

Supplementary Information

Pt size-dependent reverse oxygen spillover on Sn-doped Pt/TiO_2 for CO oxidation

Shangchao Xiong[1,2,3#], Zhengjun Gong[2#], Houlin Wang[1,2], Jianqiang Shi[1], Haiyan Liu[1], Xiaoping Chen[1], Jinxing Mi[1], Jianjun Chen[1*], Junhua Li[1]

[1]*State Key Joint Laboratory of Environment Simulation and Pollution Control, School of Environment, Tsinghua University, Beijing 100084, China*

[2]*Sichuan International Science and Technology Cooperation base for Intelligent Environmental Protection and Sustainable Development in Rail Transit*, *School of Environmental Science and Engineering, Southwest Jiaotong University, Chengdu 610031, China*

[3]*Department of Civil and Environmental Engineering, The Hong Kong Polytechnic University, Hung Hom, Kowloon, Hong Kong 100872, China*

[#]**Author contributions**

These authors contributed equally.

***Corresponding author.**

Phone: +86 010 62771093

Email address: chenjianjun@tsinghua.edu.cn

Content including: 4 Notes, 4 Tables and 17 Figures

Supplementary Note 1. The exposed crystal facets of Pt/STO catalysts

The results in Fig. 1b indicate that Pt in the 1.0Pt/STO catalyst primarily exists in the form of nanocrystals. Therefore, the most pronounced Pt aggregation site in the 1.0Pt/STO catalyst was selected to observe the exposed crystal facets of the nanocrystal Pt. As shown in Supplementary Fig. S3, the original AC-HADDF-STEM image was processed by lowering the γ value to identify the brightest region in the processed image, representing the area with the most significant Pt aggregation (highlighted with an orange rectangle). Upon magnification of this region, the interplanar spacing of the nanocrystal Pt was measured to be approximately 0.218 nm, corresponding to the (110) crystal plane of PtO (JCPDS: #43-1100). The observed (110) plane is parallel to the particle surface, suggesting that it could represent one of the major exposed facets of PtO.

Supplementary Fig. S4 shows that the support structures of all Pt/STO catalysts are rutile-phase TiO_2, with the primary exposed crystal planes being (110), $(\bar{1}10)$, and $(1\bar{1}0)$. Since rutile TiO_2 belongs to the tetragonal crystal system, these planes are equivalent, indicating that the STO support in all Pt/STO catalysts predominantly exposes the (110) crystal plane. In 0.25Pt/STO, 0.5Pt/STO, and 1.0Pt/STO, this plane is parallel to the interface of the STO support particles. This indicates that the (110) plane is one of the major exposed facets of the STO support. The study of these exposed crystal planes provides theoretical guidance for constructing structural models used in subsequent DFT simulations.

Supplementary Note 2. Absence of heat and mass transfer limitations

The absence of heat and mass transfer limitations were estimated by Mears criterion[1].

The data for the 0.5Pt/STO catalyst, which exhibited the highest CO oxidation activity, was used in the following calculations.

First, we ensured the plug-flow behavior by the following relations:

$$\frac{d_t}{d_p} = \frac{6\times10^{-3}m}{337.5\times10^{-6}m} \approx 18 > 10 \quad \text{(S1)}$$

$$\frac{L_b}{d_p} = \frac{2\times10^{-2}m}{337.5\times10^{-6}m} \approx 59 > 50 \quad \text{(S2)}$$

where d_t– reactor tube diameter, d_p – particle diameter, L_b – bed length.

Mears criterion for the interphase heat transfer limitations:

$$\frac{|\Delta H_R| r_{obs} R_p E_a}{R_g h T_b^2} < 0.15 \quad \text{(S3)}$$

where ΔH_R – reaction heat (J mol^{-1}), r_{obs} – rate per catalyst volume (mol m_{cat}^{-3} s^{-1}), R_p – particle radius (d_p/2), E_a – activation energy (J mol^{-1}), R_g – ideal gas constant (J mol^{-1} K^{-1}), h – gas-solid heat transfer coefficient (W m^2 K^{-1}), T_b – temperature of bulk fluid (K).

$$h = \frac{2\lambda_g}{d_p} \quad \text{(S4)}$$

where λ – thermal conductivity of the gas,

$$\lambda_g = y_{CO}\lambda_{CO} + y_{O_2}\lambda_{O_2} + y_{N_2}\lambda_{N_2} = 0.01(0.030) + 0.01(0.032) + 0.98(0.030) = 0.03\frac{W}{m\,K} \quad \text{(S5)}$$

$$giving\ h = \frac{2\times0.03}{337.5\times10^{-6}} = 178\frac{W}{m^2\,K} \quad \text{(S6)}$$

ΔH_R = 283×10^3 J mol^{-1}; r_{obs} = 7.08 mol m_{cat}^{-3} s^{-1}; R_p = 0.5×337.5×10^{-6} m; E_a = 35.9×10^3 J mol^{-1}; R_g = 8.314 J mol^{-1} K^{-1}; T_b = 373 K; The apparent (poured) density of the catalyst (sieved fraction): ρ_c = 1.35×10^6 g m^{-3}.

Mears criterion at 100 °C:

$$\frac{|\Delta H_R| r_{obs} R_p E_a}{R_g h T_b^2} = \frac{283\times10^3\times7.08\times\frac{337.5\times10^{-6}}{2}\times35.9\times10^3}{8.314\times178\times373^2} \approx 0.06 < 0.15 \quad \text{(S7)}$$

Therefore, our experimental conditions satisfy the Mears criterion for the absence of heat transfer limitations.

Mears criterion for the mass transport limitations:

$$\frac{r_{obs} R_p}{k_c C_{Ab}} < 0.15 \quad \text{(S8)}$$

k_c – mass transfer coefficient of the reactant (m s^{-1}), C_{Ab} – concentration of the reactant in the bulk gas phase (mol m^{-3}). Since Re << 1,

$$k_c \sim \frac{2D_{CO-N_2}}{d_p} \quad \text{(S9)}$$

$D_{CO\text{-}N2}$ – diffusion coefficient of CO in N_2 (m^2 s^{-1}).

$$k_c = \frac{2\times3.04\times10^{-5}}{337.5\times10^{-6}} \approx 0.18\frac{m}{s} \quad \text{(S10)}$$

C_{Ab} = 0.33 mol m^{-3}.

$$\frac{r_{obs}R_p}{k_cC_{Ab}} = \frac{7.08\times\frac{337.5\times10^{-6}}{2}}{0.18\times0.33} \approx 0.02 < 0.15 \quad \text{(S11)}$$

Therefore, our experimental conditions satisfy the Mears criterion for the absence of external mass transfer limitations.

Experimental diagnostic tests

To validate that the catalytic data was obtained in the kinetic regime, we performed additional diagnostic tests using our most active catalyst 0.5Pt/STO. We varied the amount of catalyst loaded in the reactor and the overall flow, keeping the ratio between the flow rate and catalyst weight constant. As shown in Supplementary Fig. S8, the conversion rate profiles are nearly identical, meaning that the reaction kinetics is not affected by the transport phenomena.

Supplementary Note 3. Details of DFT simulation

All the DFT and AIMD simulations were performed using Vienna Ab-initio Simulation Package (VASP) with projected augmented wave (PAW). The Perdew-Burke-Ernzerhof (PBE) function was applied to the generalized gradient approximation combined with the Hubbard model (GGA+U), which was based on a plane wave basis set with a kinetic energy of 400 eV. The U_{eff} (i.e., U−J) of Ti[2], Sn[3], and Pt[4-6] were set at 4.5, 5.0, and 9.0 eV, respectively. The gamma point (i.e., 1×1×1) of the K-point was employed due to the large sizes of these configurations.

The SCF tolerance and maximum atomic force were set at 10^{-6} eV and 0.05 eV/Å during geometrical optimization. All the transition states (TS) were roughly estimated by the climbing image-nudged elastic band method (CI-NEB) using 0.5 eV/Å of maximum atomic forces and a 10^{-5} eV of SCF tolerance. Then, the roughly obtained structure was used as the initial state, and then it was precisely calculated by Dimer method using 0.05 eV/Å of maximum atomic forces and 10^{-7} eV of SCF tolerance.

All AIMD simulations were conducted by sampling the canonical ensemble with Nose–Hoover thermostats and a time step of 2 fs. Because of the relatively short time scale of AIMD (10–20 ps in this study), the sampling of AIMD was only applied to extremely fast events of low energy barrier, and the observation of slow processes was excluded. Hence a large number of phase spaces could be explored quickly by enhancing the simulation temperature, and thus the AIMD simulation temperature was set at 700 K, slightly lower than the temperature adopted for the calcination of the samples[7].

Supplementary Note 4. Configurations of Pt_{sin}/STO, Pt_{clu}/STO and Pt_{cry}/STO

Ab initio molecular dynamics (AIMD) simulations combined with DFT structural optimization were employed to construct reasonable structural models for single-atom Pt, nanocluster Pt, and nanocrystal Pt supported on the STO support (denoted as Pt_{sin}/STO, Pt_{clu}/STO, and Pt_{cry}/STO) for subsequent DFT simulations. The model construction process is illustrated in Supplementary Fig. S12:

1. *In situ* NAP-XPS studies (Fig. 2) indicate that Pt on the Pt/STO catalyst surface predominantly exists in the form of PtO. Therefore, single-atom Pt (Pt_{sin}) and nanocluster Pt (Pt_{clu}) were modeled using PtO and Pt_4O_4 clusters, respectively. AC-HADDF-STEM images (Supplementary Fig. S3) show that the crystalline PtO mainly exposed (110) plane, thus the (110) plane of PtO unit cell was used to construct the heterojunction with the support to simulate nanocrystal Pt (Pt_{cry}).

2. AC-HADDF-STEM images (Supplementary Fig. S4) show that the (110) plane of rutile TiO_2 is the main exposed facet of STO. Thus, the (110) plane of rutile TiO_2 was selected as the support, with a [5×3] supercell and three stoichiometric TiO_2 layers (approximately 15 Å × 20 Å × 9 Å). Additionally, Sn was randomly substituted for 20% of the Ti atoms to construct the STO support structure.

3. After incorporating the active sites and support, the initial structures of Pt_{sin}/STO, Pt_{clu}/STO, and Pt_{cry}/STO were optimized by VASP. Subsequently, the obtained Pt_{sin}/STO, Pt_{clu}/STO, and Pt_{cry}/STO structures underwent a 10 ps of AIMD simulation at 700 K (Supplementary Fig. S13).

4. The configurations at 2, 4, 6, 8, and 10 ps of the AIMD simulation were selected as initial structures and further optimized using VASP to obtain the most stable configurations of Pt_{sin}/STO, Pt_{clu}/STO, and Pt_{cry}/STO (Supplementary Table S3).

5. The resulting configurations with the lowest energy for Pt_{sin}/STO, Pt_{clu}/STO, and Pt_{cry}/STO (Supplementary Fig. S14) were used for subsequent DFT simulation studies.

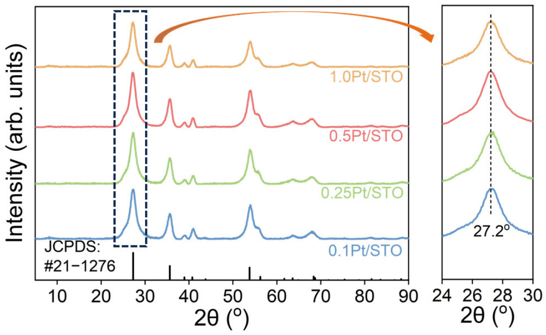

Supplementary Fig. S1 XRD spectra of Pt/STO catalysts.

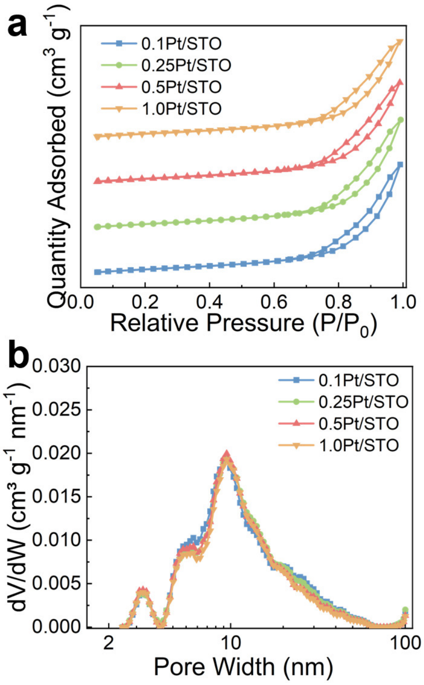

Supplementary Fig. S2 BET and BJH of Pt/STO catalysts. (a) N_2 adsorption-desorption isotherms. (b) Pore size distribution.

Supplementary Table S1 BET specific surface area (S_{BET}), total pore volume (V_p), and average pore width (W_p) of Pt/STO catalysts.

Sample	S_{BET} ($m^2\ g^{-1}$)	V_p ($cm^3\ g^{-1}$)	W_P (nm)
0.1Pt/STO	78	0.32	7.8
0.25Pt/STO	77	0.32	7.7
0.5Pt/STO	76	0.30	7.4
1.0Pt/STO	73	0.28	7.4

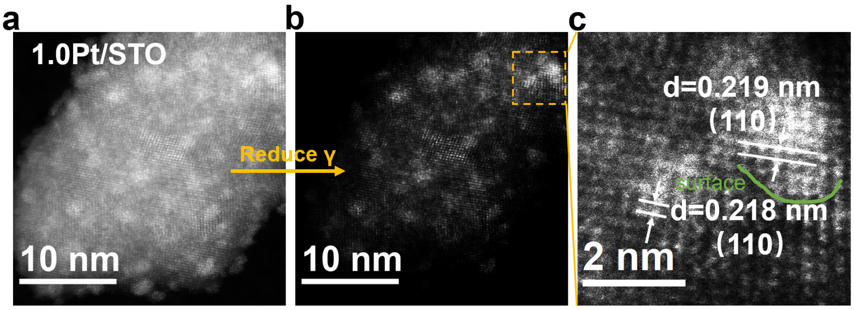

Supplementary Fig. S3 Exposed crystal facet of crystalline PtO in 1.0Pt/STO catalyst over AC-HADDF-STEM images. (a) Original image. (b) Image with reduced γ value. (c) Enlarged view.

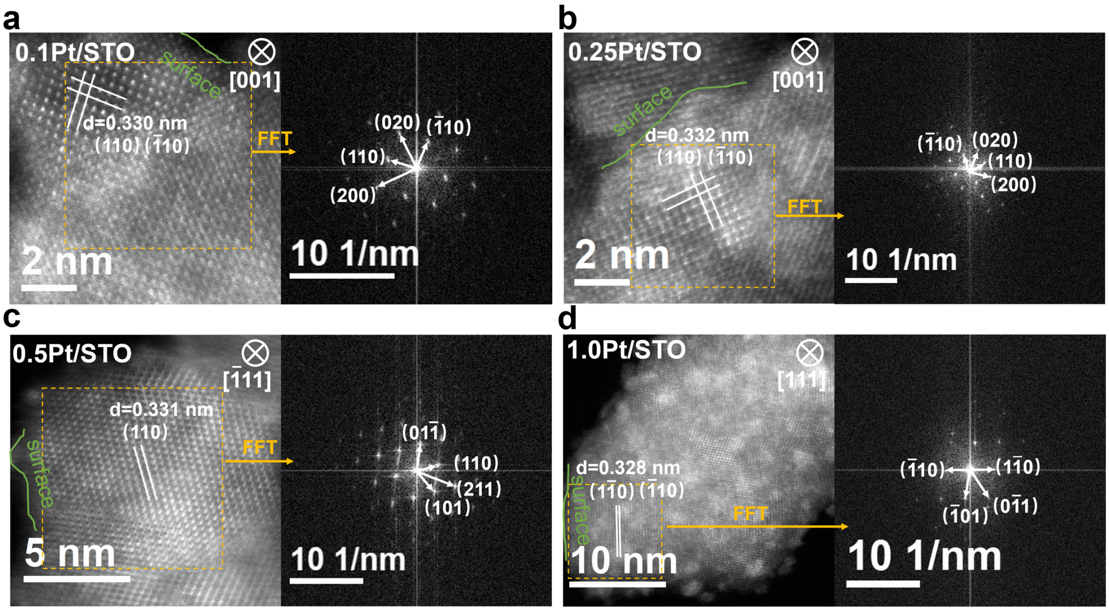

Supplementary Fig. S4 Exposed crystal facets of STO support of Pt/STO catalysts over AC-HADDF-STEM images. (a) 0.1Pt/STO. (b) 0.25Pt/STO. (c) 0.5Pt/STO. (d) 1.0Pt/STO.

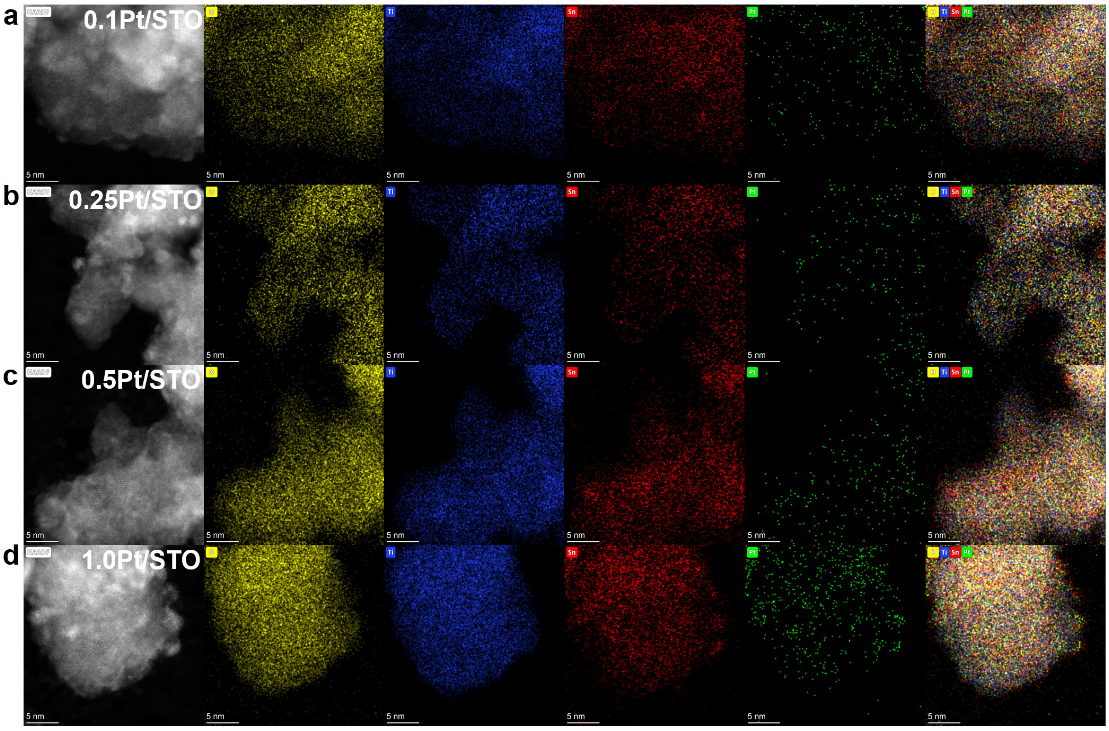

Supplementary Fig. S5 AC-STEM EDS elemental mapping of Pt/STO catalysts. (a) 0.1Pt/STO. (b) 0.25Pt/STO. (c) 0.5Pt/STO. (d) 1.0Pt/STO.

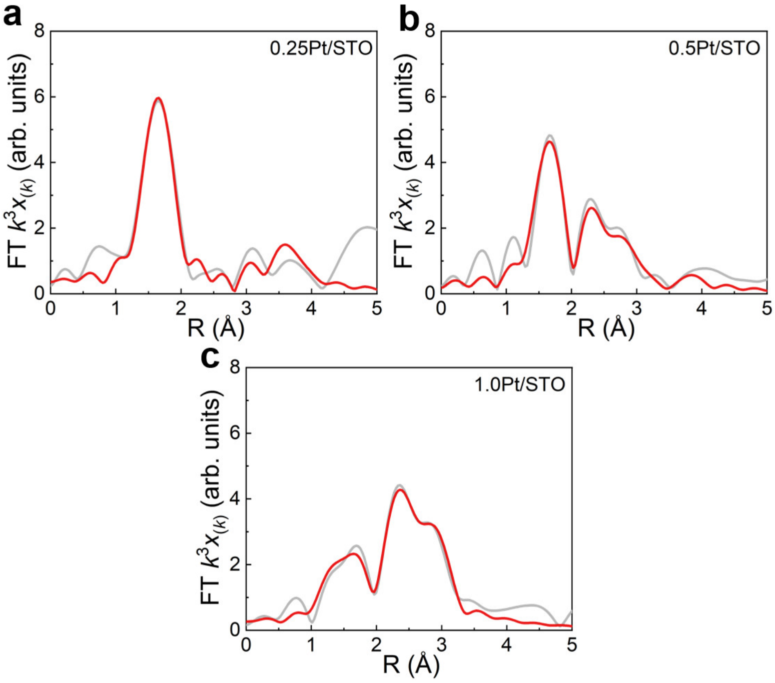

Supplementary Fig. S6 Raw and fit curves of the k^3 weighted FT-EXAFS data of Pt L_3-edge over Pt/STO catalysts. (a) 0.25Pt/STO. (b) 0.5Pt/STO. (c) 1.0Pt/STO. Gray and red lines represent the raw and fit curves, respectively.

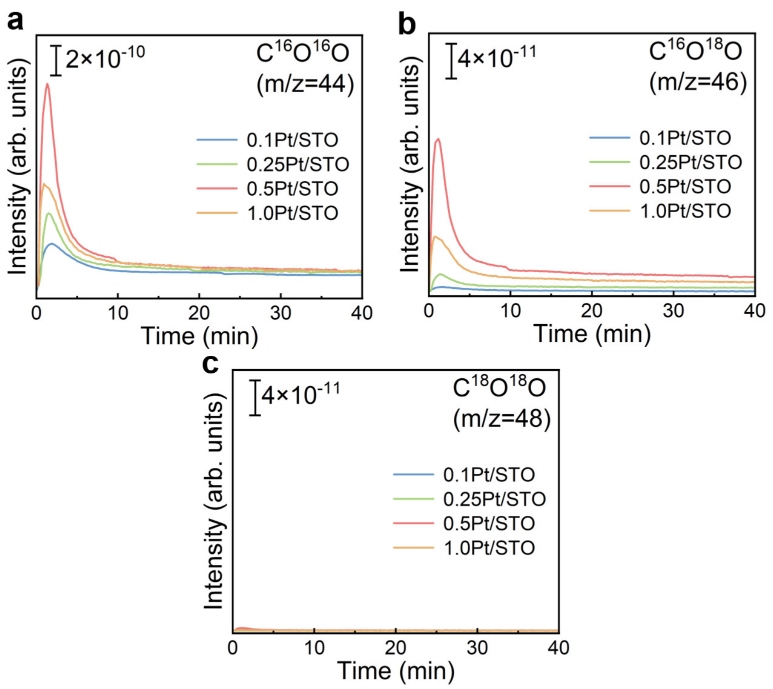

Supplementary Fig. S7 Mass spectrometry signals. (a) $C^{16}O^{16}O$, (b) $C^{16}O^{18}O$ and (c) $C^{18}O^{18}O$ produced during the flow of 1% CO/N_2 at 100 °C over Pt/STO catalysts pretreated with $^{18}O_2$ at 500 °C for 1 h.

Supplementary Table S2 EXAFS fitting results of Pt L_3-edge over Pt/STO catalysts.

	shell	CN	R(Å)	σ^2	ΔE_0	R factor
**Pt foil**	Pt-Pt	12	2.766±0.006	0.0044	8.4±1.0	0.0116
**0.25Pt/STO**	Pt-O	4.6±0.5	2.026±0.011	0.0070	15.7±1.2	0.0187
	Pt-O-Sn	1.2±0.6	3.704±0.026	0.0015		
**0.5Pt/STO**	Pt-O	3.0±0.3	2.021±0.018	0.0054	15.1±2.2	0.0331
	Pt-Pt	2.6±0.8	2.775±0.024	0.0012		
	Pt-O-Pt	1.2±0.9	2.963±0.047	0.0006		
	Pt-O-Sn	1.2±0.4	3.649±0.023	0.0055		
**1.0Pt/STO**	Pt-O	3.0±0.8	1.952±0.019	0.0128	8.1±1.5	0.0087
	Pt-Pt	6.4±1.2	2.768±0.009	0.0062		

CN: coordination numbers; *R*: bond distance; σ^2: Debye-Waller factors; ΔE_0: the inner potential correction. *R* factor: goodness of fit. S_0^2 was set to 0.76, according to the experimental EXAFS fit of Pt foil reference by fixing CN as the known crystallographic value.

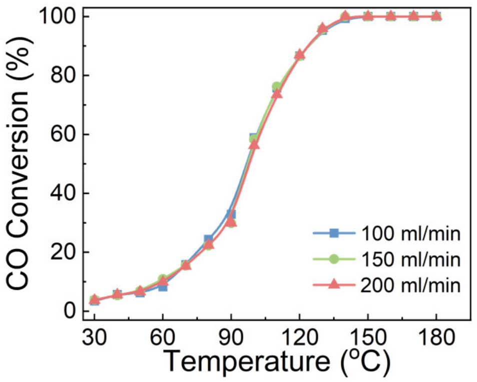

Supplementary Fig. S8 CO oxidation activity of 0.5Pt/STO at fixed space velocity. The reaction feed was 1% CO + 1% O_2 in N_2 flow. The overall flow was varied from 100 to 200 mL/min, with the catalyst weight adjusted accordingly to maintain a GHSV of 60 000 mL g_{cat}^{-1} h^{-1}.

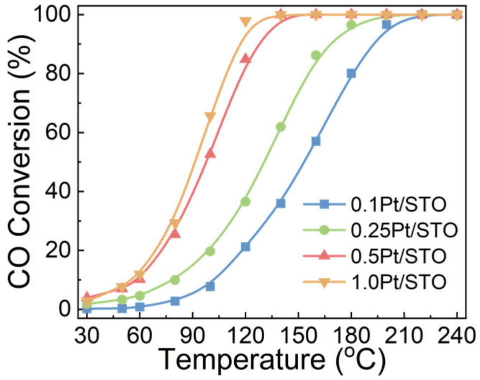

Supplementary Fig. S9 CO conversion of Pt/STO catalysts during the oxidation reaction.

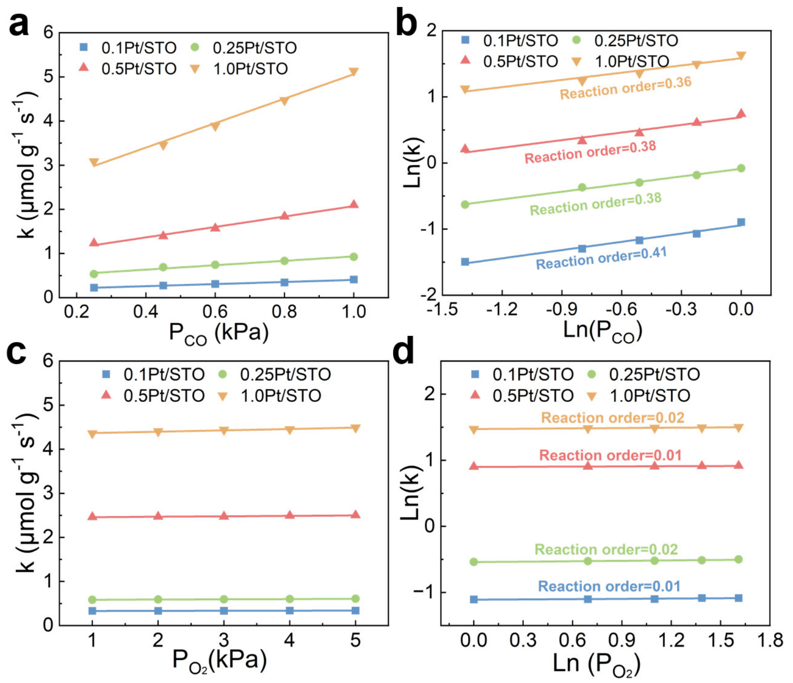

Supplementary Fig. S10 Reaction orders. CO oxidation rates as a function of (a) O_2 and (c) CO partial pressure; natural logarithm of CO oxidation rates as a function of natural logarithm of (b) O_2 and (d) CO partial pressure.

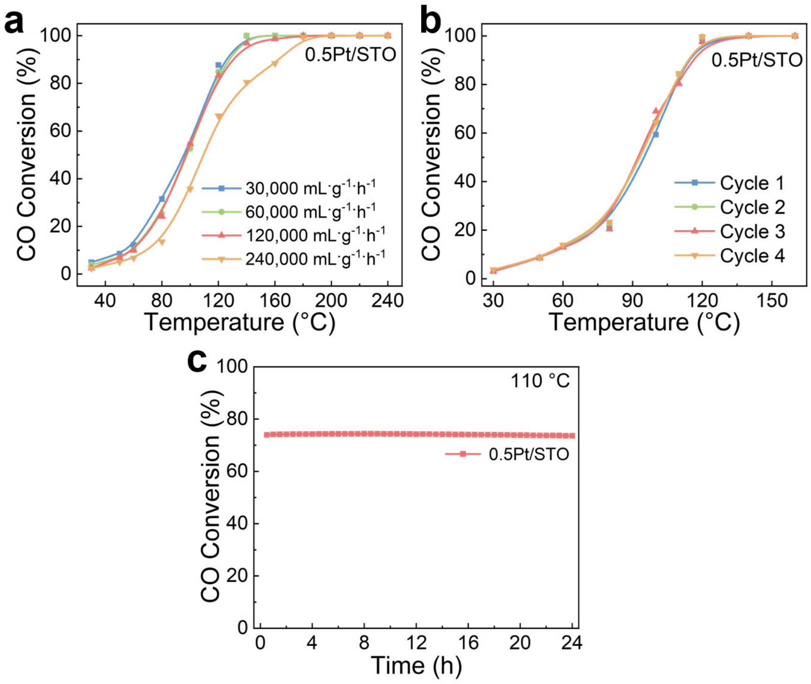

Supplementary Fig. S11 Reaction activity. (a) CO oxidation activity of 0.5Pt/STO at different GHSV. (b) Cycling performance of CO oxidation over 0.5Pt/STO. (c) 24 h stability of CO oxidation at 110 °C over 0.5Pt/STO.

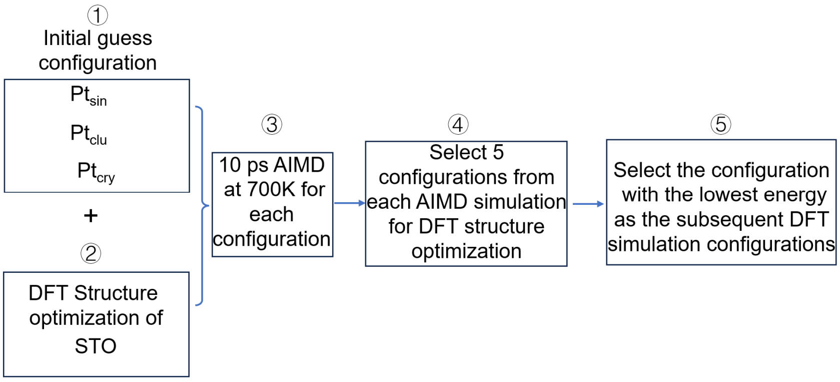

Supplementary Fig. S12 Schematic diagram of the process for generating configurations of Pt$_{sin}$/STO, Pt$_{clu}$/STO, and Pt$_{cry}$/STO catalysts for DFT simulations.

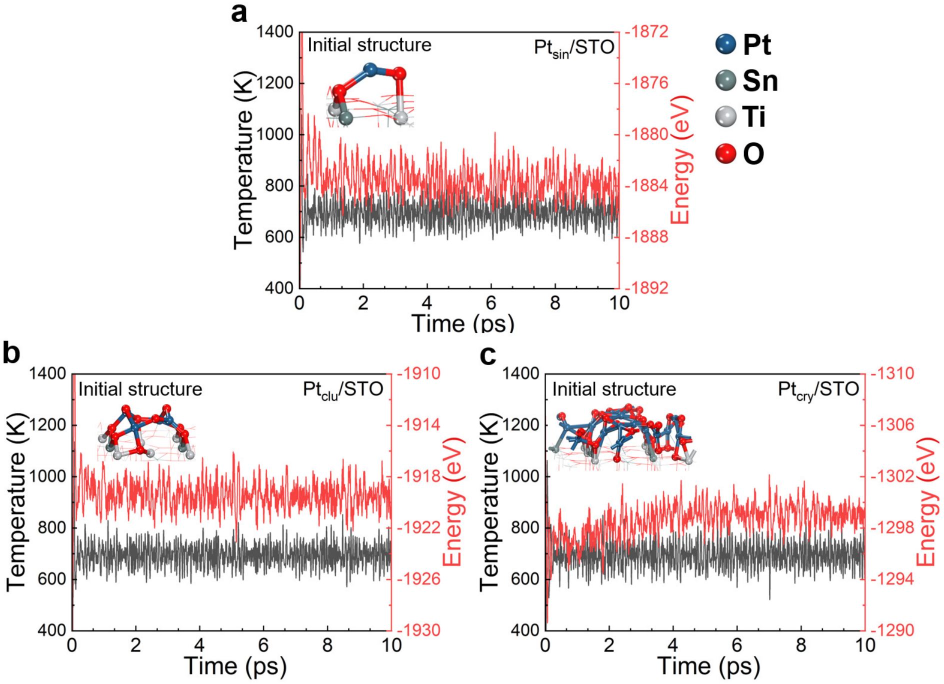

Supplementary Fig. S13 AIMD simulations. Temperature and energy profiles of (a) Pt$_{sin}$/STO, (b) Pt$_{clu}$/STO, and (c) Pt$_{cry}$/STO during 10 ps AIMD simulations at 700 K.

Supplementary Table S3 Total energy optimized by VASP using the initial configurations taken from 2–10 ps of AIMD simulation. The configuration with the lowest energy was selected to undergo DFT simulation process.

Sample	2 ps/eV	4 ps/eV	6 ps/eV	8 ps/eV	10 ps/eV	Selected configuration
Pt_{sin}/STO	-1900.64	-1900.59	-1900.59	-1900.58	-1900.59	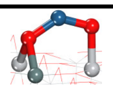 2 ps
Pt_{clu}/STO	-1936.91	-1937.07	-1937.07	-1937.06	-1937.07	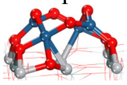 6 ps
Pt_{cry}/STO	-1460.17	-1461.37	-1461.43	-1461.38	-1461.21	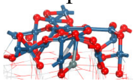 6 ps

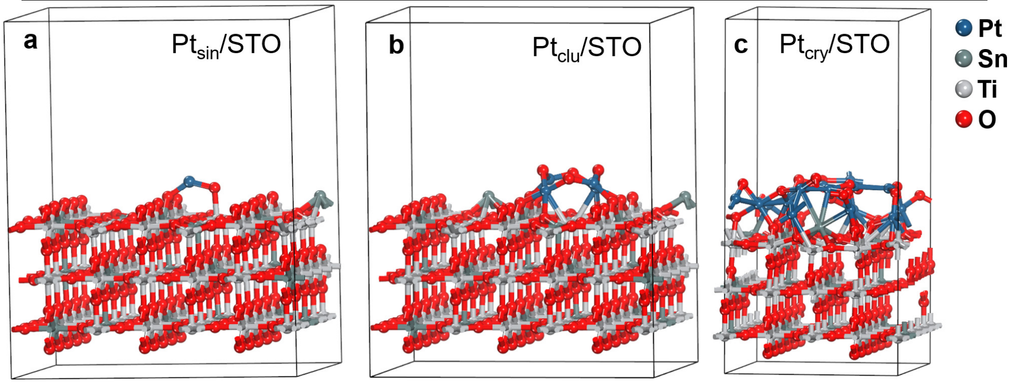

Supplementary Fig. S14 Complete configurations. The configurations of (a) Pt_{sin}/STO, (b) Pt_{clu}/STO, and (c) Pt_{cry}/STO for DFT simulations.

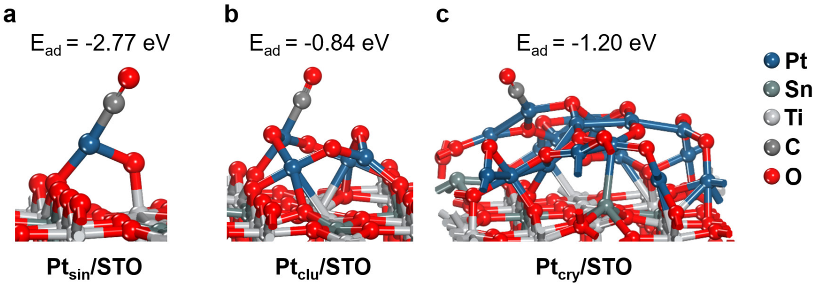

Supplementary Fig. S15 CO adsorption simulations. Configurations and adsorption energies of CO adsorbed on (a) Pt_{sin}/STO, (b) Pt_{clu}/STO, and (c) Pt_{cry}/STO.

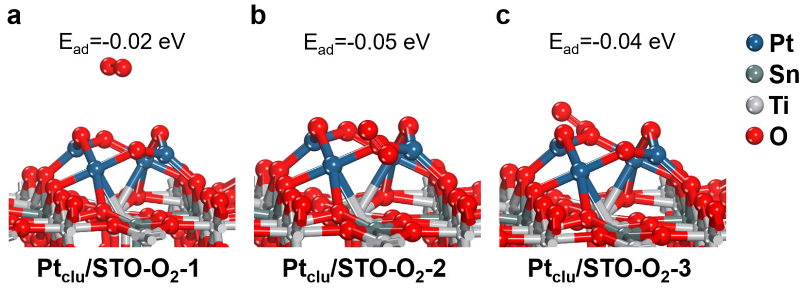

Supplementary Fig. S16 Three configurations and adsorption energies of O_2 adsorbed on Pt_{clu}/STO. (a) Pt_{clu}/STO-O_2-1. (b) Pt_{clu}/STO-O_2-2. (c) Pt_{clu}/STO-O_2-3.

Supplementary Table S4 Bader charges and charge transfer (△Q) of CO and the adsorption sites on Pt_{sin}/STO, Pt_{clu}/STO, and Pt_{cry}/STO before and after adsorption.

Sample	Before CO adsorption				After CO adsorption				△Q			
	CO		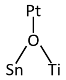		CO		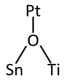		CO		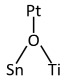	
	C	O	Pt	O	C	O	Pt	O	C	O	Pt	O
Pt_{sin}/STO	1.097	-1.097	0.977	-0.902	1.061	-1.012	1.018	-1.029	-0.036	0.085	0.041	-0.062
Pt_{clu}/STO	1.088	-1.088	0.964	-0.946	1.116	-1.015	0.955	-1.043	0.028	0.073	-0.009	-0.097
Pt_{cry}/STO	1.077	-1.077	0.479	-0.994	1.033	-1.037	0.550	-0.996	-0.044	0.040	0.071	-0.002

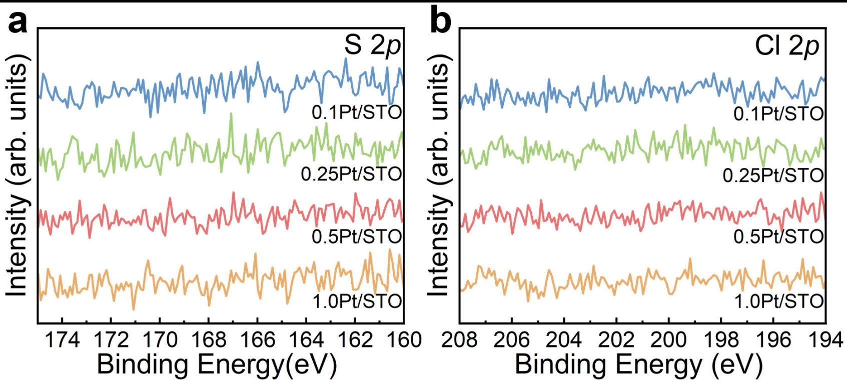

Supplementary Fig. S17 XPS spectra of Pt/STO catalysts. (a) S 2*p*. (b) Cl 2*p*.

Supplementary References

1. Muravev, V. *et al.* Interface dynamics of Pd–CeO_2 single-atom catalysts during CO oxidation. *Nat. Catal.* **4**, 469-478 (2021).
2. Park, J. B. *et al.* High catalytic activity of Au/CeO_x/TiO_2(110) controlled by the nature of the mixed-metal oxide at the nanometer level. *P. Natl. Acad. Sci. USA* **106**, 4975-4980 (2009).
3. Kandasamy, M. *et al.* Experimental and theoretical investigation of the energy-storage behavior of a polyaniline-linked reduced-graphene-oxide–SnO_2 ternary nanohybrid rlectrode. *Phys. Rev. Appl.* **14**, 024067 (2020).
4. Uddin, J., Peralta, J. E. & Scuseria, G. E. Density functional theory study of bulk platinum monoxide. *Phys. Rev. B* **71**, 155112 (2005).
5. Seriani, N., Jin, Z., Pompe, W. & Ciacchi, L. C. Density functional theory study of platinum oxides: From infinite crystals to nanoscopic particles. *Phys. Rev. B* **76**, 155421 (2007).
6. Nomiyama, R. K., Piotrowski, M. J. & Da Silva, J. L. F. Bulk structures of PtO and PtO_2 from density functional calculations. *Phys. Rev. B* **84**, 100101 (2011).
7. Wang, Y. G., Mei, D., Glezakou, V. A., Li, J. & Rousseau, R. Dynamic formation of single-atom catalytic active sites on ceria-supported gold nanoparticles. *Nat. Commun.* **6**, 6511 (2015).
